# Cytokines for evaluation of chronic inflammatory status in ageing research: reliability and phenotypic characterisation

**DOI:** 10.1186/s12979-019-0151-1

**Published:** 2019-05-21

**Authors:** Liselot Koelman, Olga Pivovarova-Ramich, Andreas F. H. Pfeiffer, Tilman Grune, Krasimira Aleksandrova

**Affiliations:** 1 Senior Scientist Group Nutrition, Immunity and Metabolism, Department of Nutrition and Gerontology, German Institute of Human Nutrition Potsdam-Rehbruecke, Arthur-Scheunert-Allee 114-116, 14558 Nuthetal, Germany; 2 University of Potsdam, Institute of Nutritional Science, Potsdam, Germany; 3 Department of Clinical Nutrition, German Institute of Human Nutrition Potsdam-Rehbruecke, Nuthetal, Germany; 4 German Center for Diabetes Research (DZD), Neuherberg, Germany; 5 Department of Endocrinology, Diabetes and Nutrition, Campus Benjamin Franklin, Charité University Medicine, Berlin, Germany; 6 Department of Molecular Toxicology, German Institute of Human Nutrition Potsdam-Rehbruecke, Nuthetal, Germany; 7 Senior Scientist Group Molecular Nutritional Medicine, Department of Molecular Toxicology, German Institute of Human Nutrition Potsdam-Rehbruecke, Nuthetal, Germany

**Keywords:** Reliability, Cytokines, Multiplex platforms, Inflammaging, Biomarkers, Ageing, BMI

## Abstract

**Background:**

There is a growing interest in the role of inflammageing for chronic disease development. Cytokines are potent soluble immune mediators that can be used as target biomarkers of inflammageing; however, their measurement in human samples has been challenging. This study aimed to assess the reliability of a pro- and anti-inflammatory cytokine panel in a sample of healthy people measured with a novel electrochemiluminescent multiplex immunoassay platform (Meso Scale Discovery, MSD), and to characterize their associations with metabolic and inflammatory phenotypes.

**Results:**

Overall, the majority of cytokines were above the limit of detection (in at least 85.3% of the samples). Cytokines IL-6, IL-8, TNF-α, IL-10, IL-13, and IFN-γ showed overall good to fair reliability (ICC > 0.40), whereas IL-1β, IL-2, IL-4, and IL-12p70 showed poor reliability (ICC < 0.40). The reliability estimates were not substantially influenced by participants’ age, sex, obesity and C-reactive protein (CRP) levels. As expected, cytokine concentrations were elevated with advanced age most pronouncedly for IL-6, IL-8, Il-2, IFN- γ, and TNF-α. No major associations with metabolic phenotypes were observed for most cytokines, with the exception of a positive association between IL-6 and TNF-α with body mass index and CRP (ρ: 0.36; ρ: 0.20; ρ: 0.53; ρ: 0.22, respectively), and IFN-γ and IL-10 with CRP (ρ: 0.23 and ρ: 0.19, respectively).

**Conclusions:**

Single measurements of selected cytokines using MSD platform, including IL-6, IL-8, IL-10, IL-13, TNF-α, and IFN-γ have shown to be representative of an individual’s average level over time and could be suitable for use in prospective epidemiological and clinical studies. Such studies are highly warranted to characterize associations of cytokines with phenotypes and diseases associated with ageing.

**Electronic supplementary material:**

The online version of this article (10.1186/s12979-019-0151-1) contains supplementary material, which is available to authorized users.

## Background

Inflammation has been increasingly recognized as an important pathophysiological phenomenon in ageing [1]. Two decades ago Franceschi et al. (2000) coined the term ‘inflammaging’ as a promising new field of research on the link between immunity, chronic inflammation, and ageing [2]. Since then, mounting evidence emerged to suggest an important role of inflammaging in the development of chronic diseases, such as Alzheimer’s disease, atherosclerosis, heart disease, type II diabetes, and cancer [3]. The underlying mechanisms by which inflammaging affects complex pathological changes and disease development are still not fully clarified [4].

Cytokines are potent soluble immune mediators disrupted in various disease states and their measurement could provide important insights into the pathogenesis of many age-related diseases and the role of inflammaging [5–7]. So far cytokine quantification in human circulation has been a challenge to both researchers and clinicians [8]. Commonly, cytokines exert biological effects at low pharmacological doses and circulating concentrations are below the limit of detection by commercially available assays kits. The cytokine blood levels have short half-lives and are prone to substantial variability potentially accounted for by diurnal rhythms, blood handling, processing, and storage, and assay methods [8].

Cytokines have been measured at messenger RNA (mRNA) levels using reverse transcription polymerase chain reaction (RT-PCR), and at protein levels by either cytokine bioassays or enzyme-linked immunosorbent assays (ELISA) [9]. These techniques have their drawbacks and the results obtained using commercial assays should be interpreted with caution [9]. Advances in laboratory technologies including flow cytometrics, Luminex bead-based assays [10], and planar multiplex assays allow measurement of a more comprehensive panel of cytokines in serum/plasma within a short period of time and with smaller specimen volumes [8]. Among novel multiplex platforms, the multiplex immunoassay platform (Meso Scale Discovery, MSD) represents a combination of electrochemiluminescence and patterned arrays with ultra-low detection limits. The reliability of cytokine measurements using MSD platform was evaluated in two recent studies [11, 12]; however, neither of these studies addressed feasibility of measurements in large population cohorts suited to advance research on immunity and ageing. Data from those studies was limited by patient characteristics [young men at risk for HIV infection] [11] or small sample size [*n* = 10] of the studies [12].

In large population cohort studies, recruited participants are predominantly healthy at study baseline and researchers aim to capture even subtle pathophysiological changes in inflammatory response to identify high risk individuals. Furthermore, most studies use a single blood sample assuming that a single measurement represents the individual’s long-term state of inflammation. So far, studies aimed to evaluate the temporal reliability of pro-inflammatory cytokine panels and characterise correlations with metabolic phenotypes in a predominantly healthy cohort have not been conducted.

We therefore aimed to assess the reliability of ten cytokines [interferon gamma (IFN-γ), interleukin-1beta (IL-1β), interleukin-2 (IL-2), interleukin-4 (IL-4), interleukin-6 (IL-6), interleukin-8 (IL-8), interleukin-10 (IL-10), interleukin-12p70 (IL-12p70), interleukin-13 (IL-13), and tumor necrosis factor alpha (TNF-α)] measured in human plasma using the MSD platform over a 4-month period. In secondary analyses, we characterized cross-sectional associations between cytokine concentrations and metabolic phenotypes.

## Results

Table 1 presents the baseline characteristics of study participants. In total, 124 women and 83 men were included in the study. The median age of the study participants was 55.4 years for women and 57.6 years for men. Participants had a median body mass index (BMI) of 26.1 kg/m^2^ (78% men had BMI ≥ 25.0 kg/m^2^; 50% females had BMI ≥ 25.0 kg/m^2^) and a waist circumference (WC) of 93.0 cm. Median systolic and diastolic blood pressure values were 136 mmHg and 88 mmHg, respectively. Blood samples were collected from majority of the participants (90%) after overnight fasting and from the rest, blood collection was randomly performed.

**Table 1:** Baseline characteristics of the study population, overall and by sex

	*All participants (n = 207)*	*Men (n = 83)*	*Women (n = 124)*
*Age (years)*	56.7 (53.7, 59.5)	57.6 (55.8, 60.4)	55.4 (51.5, 58.9)
Range	44.8–63.9	51.5–63.7	44.8–63.9
*BMI (kg/cm* ^*2*^ *)*	26.1 (23.3, 28.8)	27.8 (25.3, 29.5)	25.0 (22.6, 27.9)
Range	19.1–41.7	19.8–37.0	19.1–41.7
Overweight, BMI > 25 (%)	61	78	50
*Waist circumference (cm)*	93.0 (83.8, 101.8)	100.8 (96.1, 107.5)	86.3 (77.6, 93.3)
Range	68.3–126.3	79.3–126.3	68.3–115.8
*hsCRP (μg*/*mL)*	1.2 (0.7, 2.5)	1.5 (0.7, 2.9)	1.1 (0.6, 2.2)
Range	0.1–13.4	0.1–12.9	0.2–13.4
*Systolic blood pressure (mm Hg)*	136.0 (128.0, 144.0)	137.0 (130.0, 145.0)	134.8 (124.0, 142.0)
Range	100.0–206.0	100.0–206.0	100.0–163.0
*Diastolic blood pressure (mm Hg)*	88.0 (80.0, 94.0)	90.0 (85.0, 96.0)	86.0 (79.0, 92.0)
Range	62.0–120.0	62.0–120.0	67.0–106.0
*Sports in winter (h per week)*	1.0 (0, 2.5)	0.5 (0, 2.0)	1.0 (0, 3.0)
Range	0–14.0	0–12.0	0–14.0
*Sports in summer (h per week)*	1.0 (0, 3.0)	0 (0, 2.0)	1.0 (0, 3.0)
Range	0–14.0	0–12.0	0–14.0
Non-fasting (%)	10	13	8

*Abbreviations*: *BMI* Body mass index, *hsCRP* High sensitivity C-reactive protein. Values are expressed as medians (25th, 75th percentile), or percentages

Table 2 presents the intraclass correlations (ICCs) and 95% confidence intervals (CIs) depicting reliability of cytokine measurements over a 4-month period, overall and by sex. Overall, the reliability estimates ranged from good to fair with IL-6, IL-8 and TNF-α showing highest ICCs (0.60 < ICC < 0.70) followed by IL-10, IL-13 and IFN-γ (0.40 < ICC < 0.58). The results for IL-1β, IL-2, IL-4, and IL-12p70 pointed to rather poor reliability of these biomarkers (ICC < 0.40). No substantial differences in the ICC-s could be observed in men and women, despite somewhat higher ICCs could be seen for IL-10, IFN-γ, IL-2, IL-6, and IL-12p70 in women compared to men, whereas TNF-α showed higher ICC in men compared to women (Table 2).

**Table 2:** Repeated measurements of the cytokine concentrations, overall and by sex, with estimated ICCs

*Cytokines (pg/ml)*		*First measurement*		*Second measurement*		P difference*	*ICC (95% CI)*
		N	Median (IQR)	N	Median (IQR)		
*Interleukin-1beta*	ALL	79	3.12 (0.35–6.50)	82	3.00 (0.39–5.45)	0.89	0.25 (0.02, 0.44)
	Men	31	2.71 (0.35–4.42)	30	3.27 (0.23–6.25)	0.72	0.28 (−0.11, 0.57)
	Women	48	3.47 (0.44–7.18)	52	2.97 (0.53–5.18)	0.92	0.23 (−0.07, 0.47)
	*P* difference**		0.32		0.77		
*Interleukin-2*	ALL	143	2.13 (0.21–6.47)	131	1.77 (0.19–5.23)	0.72	0.32 (0.16, 0.46)
	Men	59	2.32 (0.21–6.23)	56	2.15 (0.21–4.66)	0.93	0.26 (0.00, 0.48)
	Women	84	1.67 (0.20–6.52)	75	0.56 (0.19–6.37)	0.62	0.36 (0.15, 0.54)
	*P* difference		0.82		0.83		
*Interleukin-4*	ALL	143	2.66 (1.53–5.75)	152	2.75 (1.57–5.98)	0.94	−0.18 (−0.34, −0.02)
	Men	56	2.69 (1.52–6.02)	60	2.70 (1.47–5.49)	0.78	−0.23 (−0.47, 0.02)
	Women	87	2.63 (1.53–5.61)	92	2.90 (1.65–6.15)	0.64	−0.15 (−0.35, 0.06)
	*P* difference		0.92		0.78		
*Interleukin-6*	ALL	207	0.52 (0.41–0.79)	207	0.52 (0.40–0.71)	0.06	0.60 (0.51, 0.68)
	Men	83	0.59 (0.44–0.82)	83	0.60 (0.44–0.81)	0.55	0.51 (0.34, 0.66)
	Women	124	0.50 (0.37–0.76)	124	0.47 (0.38–0.68)	0.04	0.63 (0.51, 0.72)
	*P* difference		0.02		0.001		
*Interleukin-8*	ALL	207	3.91 (3.10–4.88)	207	3.89 (2.99–5.02)	0.32	0.64 (0.55, 0.71)
	Men	83	3.96 (3.19–4.96)	83	4.19 (3.20–5.62)	0.07	0.62 (0.47, 0.74)
	Women	124	3.67 (3.03–4.73)	124	3.62 (2.81–4.74)	0.81	0.63 (0.51, 0.73)
	*P* difference		0.14		0.004		
*Interleukin-10*	ALL	207	0.25 (0.18–0.34)	207	0.25 (0.18–0.34)	0.76	0.58 (0.48, 0.66)
	Men	83	0.28 (0.19–0.36)	83	0.26 (0.19–0.35)	0.03	0.38 (0.18, 0.55)
	Women	124	0.24 (0.17–0.31)	124	0.24 (0.18–0.33)	0.13	0.72 (0.63, 0.80)
	*P* difference		0.02		0.35		
*Interleukin-12p70*	ALL	194	0.30 (0.15–6.01)	203	0.31 (0.16–6.01)	0.98	0.35 (0.22, 0.47)
	Men	78	1.90 (0.13–6.69)	81	1.06 (0.14–6.00)	0.29	0.27 (0.05, 0.46)
	Women	116	0.22 (0.15–5.32)	122	0.27 (0.16–6.20)	0.30	0.40 (0.24, 0.54)
	*P* difference		0.31		0.87		
*Interleukin-13*	ALL	167	0.67 (0.43–0.92)	168	0.77 (0.52–1.04)	0.01	0.43 (0.30, 0.55)
	Men	69	0.55 (0.41–0.76)	67	0.63 (0.44–0.97)	0.12	0.43 (0.21, 0.60)
	Women	98	0.75 (0.49–0.20)	101	0.83 (0.61–1.08)	0.06	0.39 (0.21, 0.54)
	*P* difference		0.01		0.01		
*Interferon-gamma*	ALL	207	3.06 (2.12–4.61)	207	3.06 (2.18–4.21)	0.43	0.40 (0.28, 0.51)
	Men	83	2.81 (2.09–4.29)	83	2.82 (2.06–3.87)	0.66	0.28 (0.07, 0.46)
	Women	124	3.37 (2.16–4.86)	124	3.26 (2.22–4.44)	0.46	0.48 (0.33, 0.61)
	*P* difference		0.28		0.14		
*Tumor Necrosis Factor alpha*	ALL	207	1.94 (1.56–2.39)	207	1.96 (1.57–2.40)	0.07	0.70 (0.63, 0.77)
	Men	83	2.04 (1.64–2.40)	83	2.14 (1.60–2.50)	0.44	0.77 (0.66, 0.84)
	Women	124	1.87 (1.51–2.36)	124	1.87 (1.56–2.33)	0.10	0.65 (0.53, 0.74)
	*P* difference		0.07		0.08		

*P value for difference based on Wilcoxon signed rank test between first and second measurements. **P value for difference based on Wilcoxon rank sum test between men and women (Kruskal Wallis) *Abbreviations*: *ICC* Intraclass correlation coefficient, *IQR* Interquartile range, *CI* Confidence interval

**Fig. 1:**
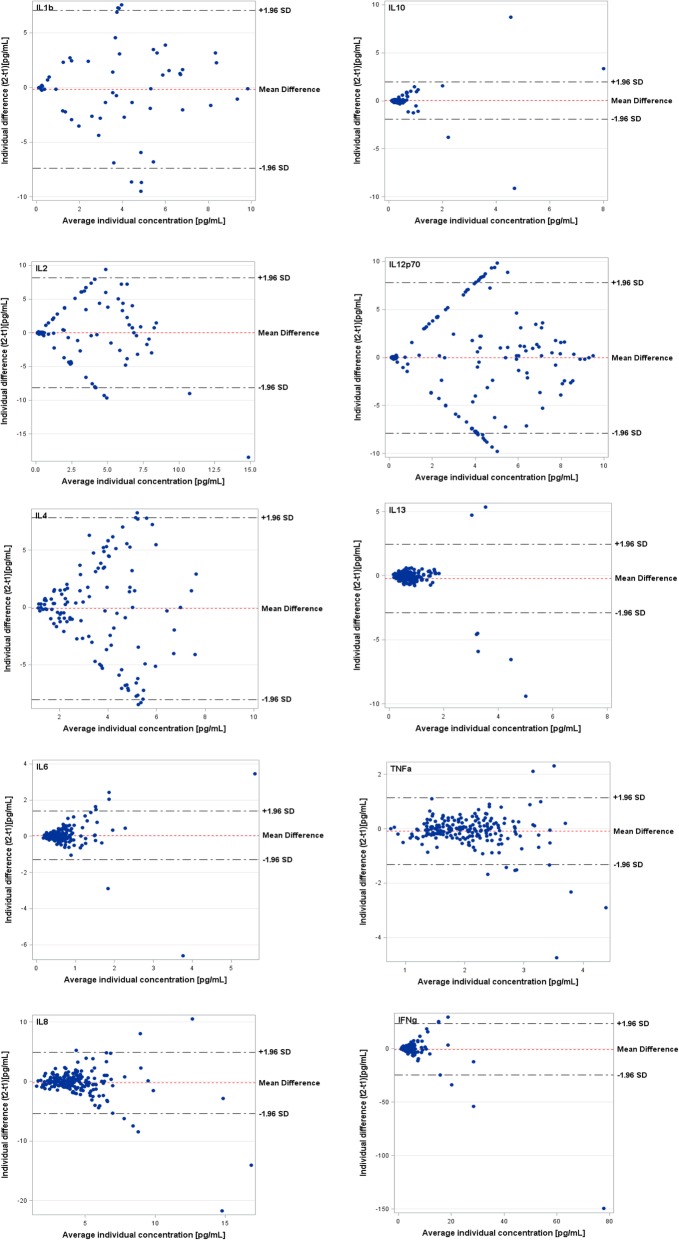
Bland-Altman plots showing the agreement between log-transformed cytokine concentrations at baseline and 4-months later in relation to average concentrations for each individual. Agreement of repeated measurements (y-axis) in relation to average concentrations (x-axis) for each individual. Horizontal lines show the mean difference and the 95% CI of limits of agreement, which are defined as the mean difference +/− 1.96 times the standard deviation of the differences

These data were additionally supported by the Bland-Altman plots that showed corresponding high levels of agreement and symmetrical distributions for cytokines with higher ICCs (TNF-α, IL-6, IL-8, IL-10, IL-13) and decreased levels of agreements and scattered distributions for those cytokines with low ICCs (IL-1β, IL-2, IL-4, IL-12p70) (Fig. 1).

**Fig. 2:**
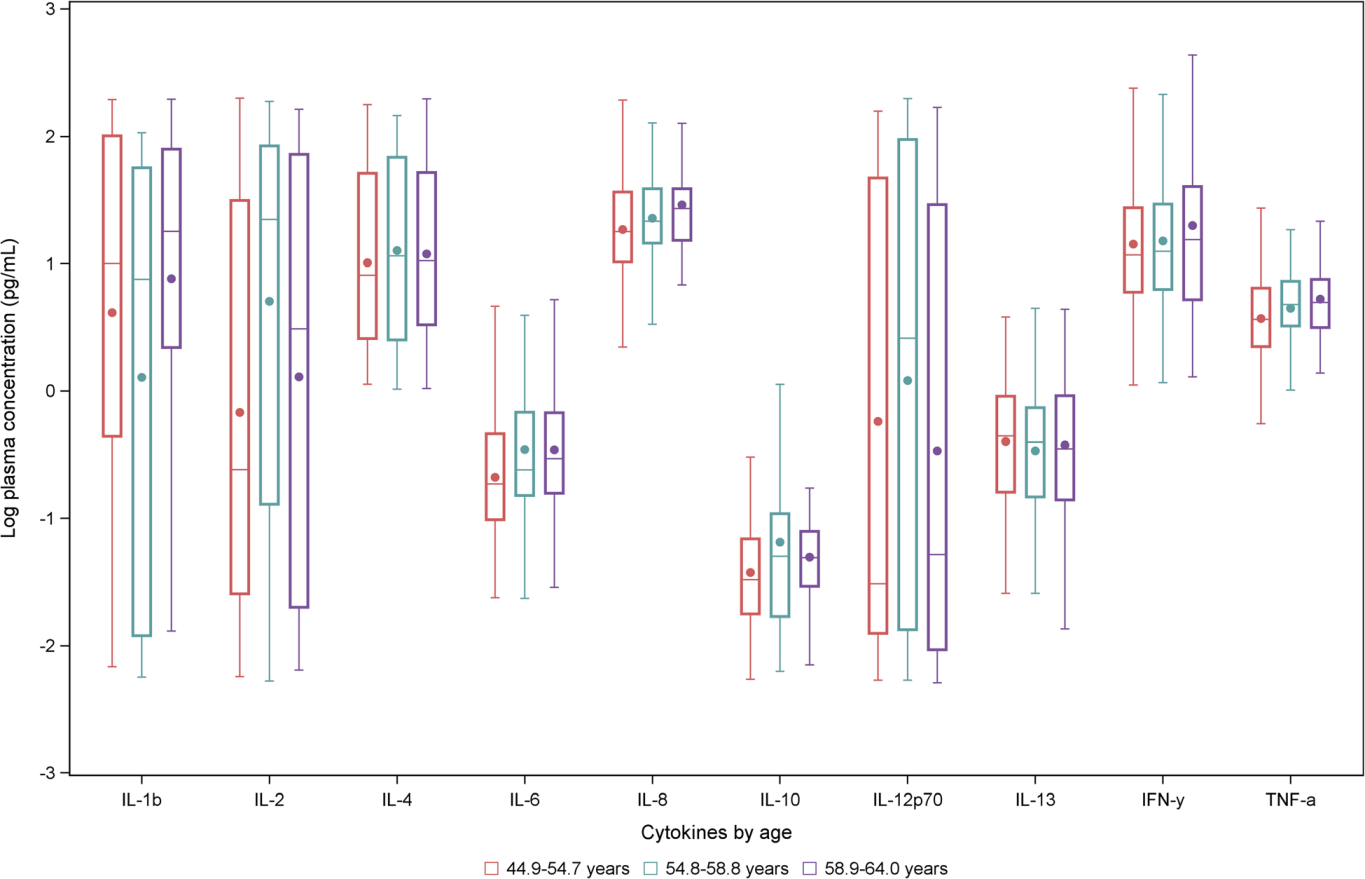
Box plots visualizing the distributions of log transformed cytokine concentrations stratified by age tertiles. This figure represents distributions of interleukin 1-beta (IL-1β), interleukin 2 (IL-2), interleukin 4 (IL-4), interleukin 6 (IL-6), interleukin 8 (IL-8), interleukin 10 (IL-10), interleukin 12p70 (IL-12p70), interleukin 13 (IL-13), interferon gamma (IFN-γ), and tumor necrosis factor alpha (TNF-α) according to increasing age categories (red: 44.9–54.7 years, green: 54.8–58.8 years, and purple: 58.9–64.0 years). Concentrations are from plasma samples collected during the first measurement

In analyses according to age categories, an increasing pattern in the median concentrations of plasma could be seen for IL-2, IL-6, IL-8, IFN-γ, and TNF-α, whereas the median concentrations of the rest of the cytokines remained almost unchanged (see Fig. 2). Table 3 presents Spearman partial correlation coefficients between mean biomarker concentrations and anthropometric parameters, high sensitivity C-reactive protein (hsCRP), and physical activity. Among all cytokines, IL-6 appeared to be most strongly associated with BMI (ρ: 0.36; 95%CI: 0.23–0.47), WC (ρ: 0.41; 95%CI: 0.28–0.51), and hsCRP (ρ: 0.53; 95%CI: 0.41–0.63). Weaker positive correlations with BMI, waist circumference, and hsCRP could also be observed with TNF-α (ρ: 0.20; 95%CI: 0.07–0.33; ρ: 0.16; 95%CI: 0.03–0.29 and ρ: 0.22; 95%CI: 0.07–0.36, respectively). An inverse albeit weak correlation was seen for IL-1β and BMI (ρ: -0.19; 95%CI: − 0.37-0.01), whereas IL-10 and IFN-γ were correlated with hsCRP (ρ: 0.19; 95%CI: 0.03–0.33 and ρ: 0.23; 95%CI: 0.08–0.37, respectively). IL-6 and TNF-α were both inversely correlated with physical activity (ρ: -0.12; 95%CI: − 0.26-0.01 and ρ: -0.15; 95%CI: − 0.28--0.02). Most of the remaining correlations were of negligible magnitude.

**Table 3:** Correlations for cytokine concentrations^a^ with BMI, WC, hsCRP, and sports adjusted for age and sex

Biomarker	BMI (kg/cm^2^)			Waist Circumference (cm)			hsCRP (ug/ml)			Reported sports activities^b^ (hours/week)		
	N	ρ^c^	95% CI^d^	N	ρ^c^	95% CI^d^	N	ρ^c^	95% CI^d^	N	ρ^c^	95% CI^d^
*IL-1β*	102	−0.19	− 0.37, 0.01	102	−0.15	− 0.33, 0.05	76	0.06	−0.17, 0.28	79	0.09	−0.14, 0.3
*P*-value		0.06			0.15			0.62			0.46	
*IL-2*	159	0.04	−0.11, 0.2	159	0.08	−0.07, 0.24	126	−0.02	− 0.2, 0.15	143	0.07	−0.1, 0.23
*P*-value		0.59			0.30			0.78			0.42	
*IL-4*	175	−0.06	−0.21, 0.09	175	−0.06	−0.21, 0.09	137	0.03	−0.14, 0.2	143	0.03	−0.14, 0.19
*P*-value		0.44			0.69			0.42			0.76	
*IL-6*	207	0.36	0.23, 0.47	207	0.41	0.28, 0.51	164	0.53	0.41, 0.63	207	−0.12	−0.26, 0.01
*P*-value		<.0001			<.0001			<.0001			0.08	
*IL-8*	207	0.05	−0.08, 0.19	207	0.04	−0.1, 0.18	164	0.06	−0.1, 0.21	207	0	−0.14, 0.14
*P*-value		0.44			0.58			0.46			0.98	
*IL-10*	207	0	−0.14, 0.13	207	0.02	−0.12, 0.15	164	0.19	0.03, 0.33	207	0.04	−0.1, 0.17
*P*-value		0.96			0.82			0.02			0.60	
*IL-12p70*	205	0.06	−0.08, 0.19	205	0.10	−0.04, 0.23	162	−0.02	− 0.17, 0.14	194	0.07	−0.08, 0.21
*P*-value		0.43			0.16			0.82			0.36	
*IL-13*	191	0.06	−0.09, 0.2	191	0.06	−0.09, 0.2	152	0.12	−0.04, 0.28	167	0.04	−0.12, 0.19
*P*-value		0.44			0.45			0.14			0.63	
*IFN-γ*	207	0.04	−0.1, 0.17	207	0.09	−0.05, 0.22	164	0.23	0.08, 0.37	207	0.01	−0.12, 0.15
*P*-value		0.60			0.22			0.004			0.84	
*TNF- α*	207	0.20	0.07, 0.33	207	0.16	0.03, 0.29	164	0.22	0.07, 0.36	207	−0.15	−0.28, −0.02
*P*-value		0.004			0.02			0.004			0.03	

^a^Mean cytokine concentrations from both measurements ^b^Sports in winter with first blood sample from October–March. ^c^Spearman partial correlation coefficient. ^d^Based on Fisher’s *z* transformation *Abbreviations*: *BMI* Body mass index, *CI* Confidence interval, *hsCRP* High sensitivity c-reactive protein, *IL-1β* Interleukin 1-beta, *IL-2* Interleukin 2, *IL-4* Interleukin 4, *IL-8* Interleukin 8, *IL-10* Interleukin 10, *IL-12p70* Interleukin 12p70, *IL-13* Interleukin 13, *IFN-γ* Interferon gamma, *IQR* Interquartile range, *TNF-α* Tumor necrosis factor alpha, *WC* Waist circumference

Finally, to facilitate application of current results for correcting measurements in future studies we provide estimates of true vs observed risk depending on the ICCs of the cytokines (Additional file 5). Differences between hypothetical risk ratios (of 1.5, 2.5, and 3.5) and observed risk ratios are caused by the (imperfect) reliability of a biomarker due to intra-individual variation. As an example, if one wants to explore exposure-outcome association of IL-8 based on a single measure, the measured IL-8 would lead to an underestimation of the true risk ratio. The observed risk ratio would for example be 2.3 whereas the true risk ratio is 3.5, taking into account the specific ICC of IL-8 (0.64).

## Discussion

In this study, conducted among predominantly healthy individuals, we evaluated the reliability of circulating concentrations of ten cytokines measured with novel MSD platform over a 4-month period of time. Overall, the majority of cytokines were above the limit of detection (in at least 85.3% of the samples). The reliability estimates ranged from good to poor. Cytokines IL-6, IL-8, TNF-α, IL-10, IL-13, and IFN-γ showed overall good reliability (ICC > 0.40), whereas IL-1β, IL-2, IL-4, and IL-12p70 showed poor reliability (ICC < 0.40). The reliability estimates were not substantially influenced by participants’ age, sex, obesity status (normal or overweight) and baseline CRP levels. Cytokine concentrations were elevated with advanced age most pronouncedly for IL-6, IL-8, IL-2, IFN-γ, and TNF-α. Furthermore, elevated concentrations of IL-6, TNF-α, IL-10, and IFN-γ were associated with overweight and obesity (BMI above 25).

Several studies have provided evidence of within-person temporal stability for panels of circulating cytokines measured using ELISA and multiplex immunoassays as shown in Table 4 [11–20]. Among these studies, the Luminex bead-based assay was the most frequently used method. Among different cytokines, the most commonly evaluated biomarkers were IL-6, TNF-α, IL-8, and IL-10. However, comparison with results from previous studies was hampered by differences in study population, time periods between measurements, biosample material and cytokine panels (Additional file 4).

**Table 4:** ICCs with 95% CIs of inflammatory cytokines in predominantly healthy participants previously published in literature

Reference	Assay	Cytokines									
		IL-1β	IL-2	IL-4	IL-6	IL-8	IL-10	IL-12p70	IL-13	IFN-γ	TNF-*α*
This study	MSD	0.25 (0.02, 0.44)	0.32 (0.16, 0.46)	−0.18 (− 0.34, − 0.02)	0.60 (0.51, 0.68)	0.64 (0.55, 0.71)	0.58 (0.48, 0.66)	0.35 (0.22, 0.47)	0.43 (0.30, 0.55)	0.40 (0.28, 0.51)	0.70 (0.63, 0.77)
McKay et al. 2017 [11]	MSD	ND	ND	–	0.60 (0.51, 0.68)	–	0.85 (0.81, 0.88)	0.84 (0.79, 0.87)	–	ND	0.54 (0.46, 0.63)
Belzeaux et al. 2017 [12]	MSD	ND	–	ND	0.04	−0.01	0.12	0.83	ND	0.07	0.74
	Luminex	0.96	–	0.90	0.95	0.89	0.96	0.89	0.90	0.97	0.96
Epstein et al. 2013 [13]	Luminex	0.49 (0.41, 0.62)	–	–	0.55 (0.47, 0.62)	0.34 (0.25, 0.43)	0.70 (0.64, 0.75)	–	–	0.55 (0.47, 0.62)	0.87 (0.84, 0.90)
Todd et al. 2013 [14]	Erenna Immunoassay System^a^	–	–	–	0.46	–			–	–	0.39
Nash et al. 2013 [15]	ELISA	–	–	–	0.72	–	–	–	–	–	–
Navarro et al. 2012 [16]	ELISA/ Luminex^b^	–	–	–	0.48 (0.36, 0.62)	0.73 (0.63, 0.83)	–	–	–	–	0.92 (0.89, 0.96)
Hofmann et al. 2011 [17]	Luminex	ND	ND	ND	0.84 (0.70, 0.91)	0.55 (0.28, 0.71)	0.60 (0.35, 0.76)	ND	0.73 (0.52, 0.85)	ND	0.86 (0.74, 0.93)
Clendenen et al. 2010 [18]	Luminex	0.73 (0.43, 0.89)	0.80 (0.56, 0.92)	0.70 (0.36, 0.87)	0.81 (0.56, 0.92)	0.86 (0.68, 0.95)	0.75 (0.46, 0.90)	0.77 (0.50, 0.91)	0.81 (0.56, 0.92)	0.72 (0.41, 0.89)	0.69 (0.36, 0.87)
Gu et al. 2009 [19]	Luminex	0.86 (0.78, 0.91)	0.81 (0.71, 0.88)	0.92 (0.87, 0.95)	0.92 (0.88, 0.95)	0.02 (0.00, 0.21)	0.75 (0.63, 0.84)	0.83 (0.74, 0.89)	–	–	0.88 (0.82, 0.92)
Lee et al. 2007 [20]	Luminex	0.77	–	–	0.73	0.51	–	–	–	–	0.48

^a^Laboratory developed tests based upon single-molecule counting technology. ^b^IL-6 was measured with ELISA, IL-8 and TNF-α with Luminex. *Abbreviations*: *ICC* Intraclass correlation coefficient, *IL-1β* Interleukin 1-beta, *IL-2* Interleukin 2, *IL-4* Interleukin 4, *IL-8* Interleukin 8, *IL-10* Interleukin 10, *IL-12p70* Interleukin 12p70, *IL-13* Interleukin 13, *IFN-γ* Interferon gamma, *MSD* Meso Scale Discovery, *TNF-α* Tumor necrosis factor alpha, *ND* Not determined for analytes with low % detected (usually < 40% samples above LLOD or high coefficients of variability of duplicates)

Recently, the MSD platform was applied in two studies aimed to evaluate the intra-individual reliability in cytokine measurements over a short and long period of time [11, 12]. Compared to our results these studies reported lower detection rates and poorer reliability estimates for IL-1β, IL-2, IL-4, and IL-13. The first study was based on 250 young men at risk for HIV infection with repeated blood sample collections over 15 years [11]. With exception for IFN-γ and IL-2 most of the other cytokines were detectable in > 80% of the samples and had fair to strong within-person correlation (ICC > 0.40) up to 15 years. For example, the ICCs for IL-6, IL-10, IL-12p70, and TNF-α from first to last samples were 0.46, 0.71, 0.73, and 0.49, respectively [11]. In the second study based on data from 10 healthy controls with repeated blood collections over a 30 week period, the ICCs for IFN-γ, IL-6, IL-8, and IL-10 were 0.07, 0.04, 0.01, and 0.11, respectively [12]. Possible reasons for the lower detection rates and differences in reliability estimates among different studies could be sought in the low circulating levels of these biomarkers in predominantly young male individuals [11], the small sample size [12], differences in short and long term time intervals or potential degradation of certain proteins during (long-term) storage [19].

Our results could guide researchers of future prospective studies of plasma cytokines to estimate the true relative risk given the observed relative risk. In particular, ICCs can be used to correct relative risks or correlation coefficients and their confidence intervals for random within-person variation to account for the attenuation introduced by measurement error [21]. Measurement error correction would have a substantial effect on the final estimate for cytokines with modest ICCs as shown for other biomarker studies [22].

A wide range of factors could affect the circulating levels of cytokines. These include age, sex, adiposity status and overall inflammatory state. We therefore evaluated whether observed results for the reliability of measured cytokines could be also influenced by some of these factors. Our data did not reveal pronounced differences by strata of age, sex, obesity and CRP levels arguing against possible influence of any of these factors on the observed reliability estimates. Differences among circulating cytokine concentrations in individuals could also exist due to seasonal, hormonal, or circadian physiological variability. Cytokine concentrations were higher in afternoon measurements compared to morning measurements, supposedly reflecting influences by circulating cortisol [23]. These findings are consistent with previous work showing daytime regulation of inflammatory mediators including IL-6 in healthy individuals [24]. In our study, however, variations might have been limited due to restricted time interval when the samples were taken.

Annual seasonality was also suggested to be an important environmental factor influencing cytokine production [25]. Seasonal variation may reflect a physiological response to changes in daylight hours, indoor or outdoor temperature, or variations in physical activity in winter and summer season. For instance, in a cohort of 530 healthy individuals the production of several cytokines (IL-1β, IL-6, and TNF-α) showed significant peaks in summer compared to other seasons [25]. As we had first and second measurements taken in different seasons, between October–March (autumn/winter) and February–July (winter/spring/summer), and we only had a single blood sample per time point, we cannot determine whether variations are dependent of seasonality. However, since we did not detect major differences between the two measurements, we could exclude major influence of seasonality on biomarker stability. Similar to our findings, a previous reliability study evaluating seasonal variability of IL-1β, IL-6, IL-8, and TNF-α (*n* = 48) observed no substantial variation over seasons [20].

While the major focus of our study was to provide a methodological basis to researchers in planning future analyses employing cytokine measurements, our data also allowed exploration of associations between the range of cytokines and individual phenotypes. Our primary interest was related to cytokines as potential biomarkers of inflammageing [26]. Higher levels of cytokines have been associated with age-related diseases, such as cardiovascular diseases and cognitive decline [27]. The systemic effects of cytokines and the complex biochemical interactions with other pathophysiological pathways have not been well described [28]. Despite the narrow age-range in our study (40–64 years), we could observe a clear trend towards increasing cytokine concentrations with increasing age that was most pronounced for IL-6, IL-8, IL-2, IFN-γ, and TNF-α (Additional file 3). Furthermore, in line with previous evidence TNF-α and IL-6 were positively correlated with BMI, WC and CRP [15, 29]. Our data further suggested a positive association between IFN-γ and IL-10 with CRP. Although IL-10 can be categorized as anti-inflammatory and CRP as pro-inflammatory, these cytokines are both activated in acute-phase inflammation and chronic inflammatory diseases. The association we found between IFN-γ and CRP is comparable with previously published work, where a proxy marker of IFN-γ production (neopterin) was positively associated with CRP and other metabolic biomarkers in a subgroup of healthy EPIC participants [30]. These results enhance our knowledge on the interplay of immune responses and metabolism. However, larger sample size studies would be needed in the future to characterize lifestyle patterns potentially associated with cytokine profiles. Whether measuring the full range of biomarkers would be useful to evaluate the role of inflammaging in epidemiological research remains questionable.

Our study has several strengths. We applied the electrochemiluminescent multiplex immunoassay platform (Meso Scale Discovery, MSD) as a new convenient technique that may be useful for future epidemiological studies employing a large number of participants. Our study population included both sexes and our sample size was relatively large for a validation study as compared to most reliability studies in the literature (see Additional file 4). The biomarkers we measured represented different aspects of immune-inflammatory pathways during adaptive and innate immune responses, having pro-inflammatory (IL-1β, IL-2, IL-8, IL-12p70, TNF-α, IFN-γ) or anti-inflammatory (IL-4, IL-10) effects, or both (IL-6, IL-13).

Several limitations of the analysis should be considered. First, the study population included predominantly healthy adult individuals living in a specific geographic area (Potsdam, Germany) which would potentially limit generalisability to other population groups. However, based on measurements of hsCRP that could be used as a proxy marker of systemic inflammatory response, the results have remained robust. Cytokine concentrations could be influenced by sampling methods and storage conditions. We have measured cytokines in plasma EDTA using samples collected several years prior measurement. Previous studies that compared results on cytokine quantification in different media, i.e. plasma versus serum, did not suggest major differences particularly at low biomarker concentrations as in our study [12, 31, 32]. Plasma samples could be more reproducible due to anticoagulants (i.e. EDTA) that control activity of the blood sample and previous validation study showed an excellent intra- and inter-assay reliability as well as robust protein recovery efficiency of MSD platform in human plasma [33]. The storage time and the number of thow-freeze cycles were also shown to influence cytokine measurements [32] and our results should be interpreted taking these factors into account. We evaluated cytokine reliability over a 4 month period and further studies suited for evaluation of long term cytokine reliability are warranted in the future.

## Conclusion

This study has provided first lines of evidence, as per our knowledge, on the reliability of cytokine concentrations measured with a novel MSD platform methodology. Our results suggested that single measurements of IL-6, IL-8, TNF-α, IL-10, IL-13, and IFN-γ could accurately assess the biomarker variability within an individual over 4 month period and could be suitable for use in prospective epidemiological and clinical studies. Such studies are highly warranted to characterize associations of cytokines with phenotypes and diseases associated with ageing.

## Methods

### Study population

**Fig. 3:**
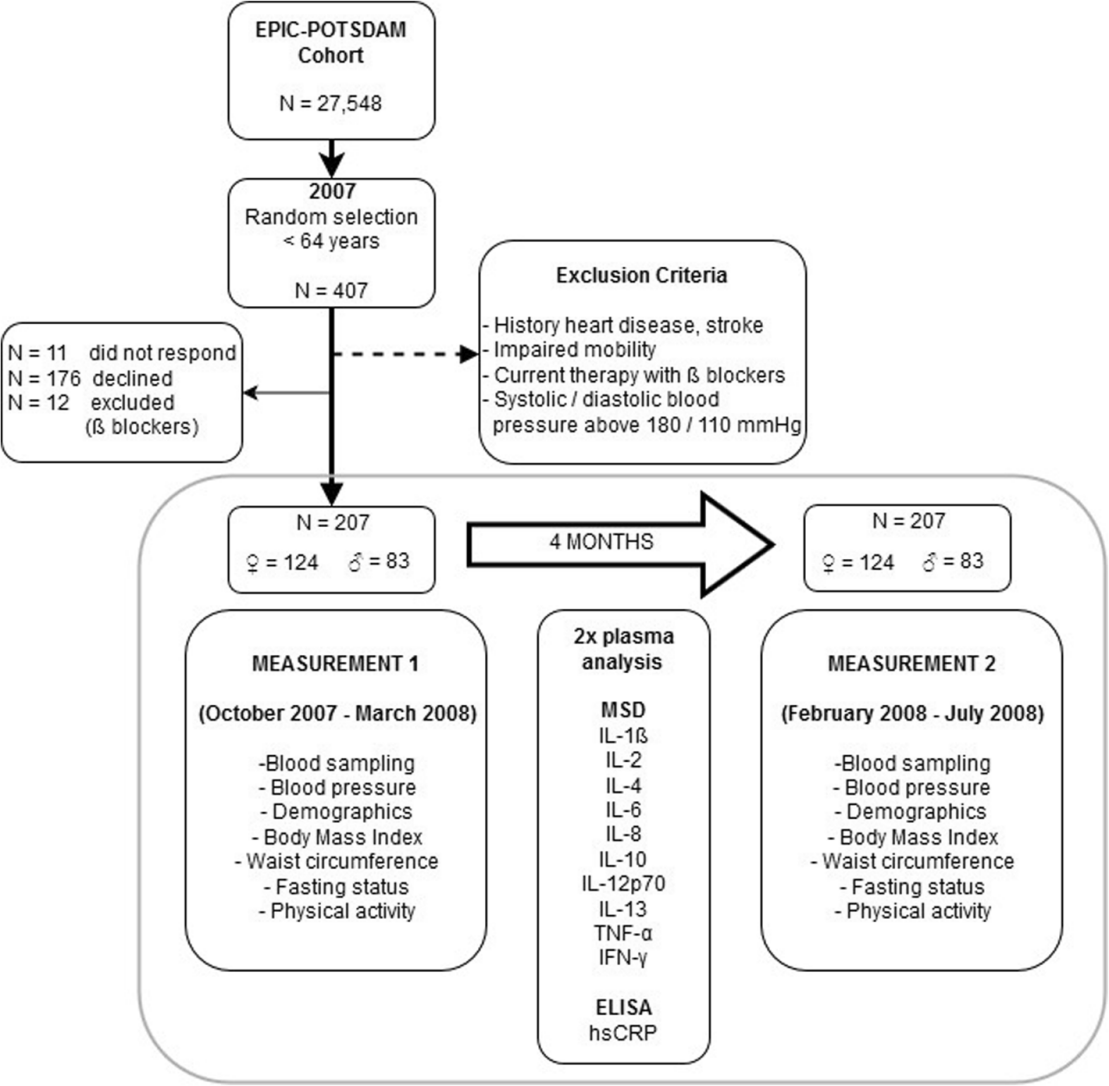
Flow diagram of the study design. A total of 207 participants (124 women and 83 men) from the EPIC-Potsdam Cohort completed this study. Single blood samples were collected on two occasions, 4 months apart

The study was based on a randomly selected analytical sample of individuals (< 64 years old) taking part in a validation study conducted within the European Prospective Investigation into Cancer and Nutrition (EPIC)-Potsdam study [34] (Fig. 3). Exclusion criteria included history of heart disease (myocardial infarction, heart failure, cardiomyopathy, stroke, angina pectoris), impaired mobility, reported use of β-blockers, and had systolic or diastolic blood pressure above 180 mmHg or 110 mmHg, respectively. Of the 407 invited participants, the total number of eligible participants with available sample collections on two occasions taken 4 months apart was 207.

The blood collection took place during weekdays in the mornings between 8 and 11 am. The first blood samples were collected between October 2007 and March 2008 and the second between February and July 2008.

Written informed consent was obtained from all participants and the Ethics Committee of the Medical Association of Brandenburg approved the study procedures.

### Laboratory analyses

Blood plasma/serum was separated and stored at − 80 °C. 50 μl of plasma EDTA were retrieved for cytokine quantification. Measurements were performed at the Department of Clinical Nutrition, German Institute of Human Nutrition Potsdam-Rehbrücke, Germany by trained laboratory staff. Measurements of hsCRP (high sensitivity C-reactive protein) were performed with commercially available sandwich ELISA (BioVendor, Kassel, Germany) according to the manufacturer’s instructions. Repeated samples of each participant were measured in the same analytical batch.

The MSD V-Plex Proinflammatory Panel 1 Human Kit (MSD platform) (Rockville, Maryland, USA) was used to measure plasma IL-1β, IL-2, IL-4, IL-6, IL-8, IL-10, IL-12p70, IL-13, IFN-γ, and TNF-α concentrations in single samples, according to the manufacturer’s instructions. Intra-class coefficients of variations were typically below 7%, and inter-class coefficients of variations were below 15%. The lower limits of detection (LLOD) for the cytokines ranged between 0.01–0.89 pg/mL. Overall, the majority of cytokines were above the limit of detection (in at least 85.3% of the samples). For IL-6, IL-8, IL-10, IFN-γ and TNF-α, IL-12p70 and IL-13 more than 80% of the measurements could be detected at both study time points (see Additional file 1). For IL-1β, IL-2, and IL-4 the average percent of detected measurement values were 38.9, 66.2, and 71.3, respectively. In one individual the difference of IFN-γ concentration between two measurements was notably large (2.99–150 pg/mL), hence reported as outlier and excluded from the analysis.

### Anthropometric measurements

Measurements of height, weight, waist circumference (WC), and systolic- and diastolic blood pressure were collected at the first and second visits. Height was measured with a rigid stadiometer; weight was measured using a standard scale or bio-impedance scale [35]. BMI (body mass index) was calculated from height and weight (kg/m^2^). Level of physical activity was assessed with a self-reported physical activity questionnaire (EPIC-PAQ) that has previously been validated in this study sample [35].

### Statistical analysis

Statistical analyses were performed using SAS software package, release 14.2 (SAS Institute, Cary, NC, USA). *P* value < 0.05 was considered statistically significant, and statistical tests used were two-sided. Variable distribution was evaluated based on quantile-quantile plots and histograms. Non-normally distributed data was transformed using the natural logarithm in order to allow parametric testing. Biomarker concentrations were presented as medians and interquartile ranges. For each biomarker, Wilcoxon signed rank test was used to compare concentrations between first and second measurements. Wilcoxon rank sum test (Kruskal Wallis) was used to compare concentrations between men and women for each measurement. As a measure of reliability between the two measurements, the intraclass correlation coefficient (ICC) was calculated for each biomarker, total and stratified by sex. ICCs were calculated by dividing the between-subject variance by the total variance (sum of between- and within-subject variances). Based on the ICC estimate, values less than 0.40, between 0.40 and 0.60, between 0.60 and 0.74, and greater than 0.75 were indicative of poor, moderate, good, and excellent reliability, respectively. To evaluate potential variability due to individual characteristics, we calculated ICCs of each cytokine according to participants’ BMI, WC, hsCRP, and age (see Additional file 2). To create respective categories, we used cut-points based on median population values, i.e. BMI, 26.1 kg/m^2^; WC, men 100.8 cm, women 86.3 cm; hsCRP, 1.2 μg/mL; age, 56.7 years. Bland-Altman plots based on the means and the standard deviations of the differences between two repeated cytokine measurements were further created [36]. Age was stratified into tertiles such that the distribution of the cytokine concentrations could be illustrated in a boxplot according to increasing age categories. Tertiles were grouped as follows: low: 44.9–54.7 years, middle: 54.8–58.8 years, high: 58.9–64.0 years. Plasma samples were used from the first measurement. Wilcoxon rank sum test (Kruskal Wallis) was used to compare concentration differences in age categories per cytokine. Correlations of biomarker concentrations with BMI, WC, hsCRP, and physical activity were evaluated using Spearman correlation analyses. Average biomarker concentrations from the first (baseline) and second (after 4 months) measurements were used for correlations with BMI, WC, and hsCRP. Physical activity during winter was correlated with the first blood sample collected during October 2007 – March 2008. Correlations were adjusted for age and sex. Fisher’s *z* transformation was used to produce 95% confidence interval (CI) for each correlation coefficient.

In order to facilitate future observational studies with application of the measured biomarkers and their ICCs, we calculated the degree of attenuation of risk estimates that arises due to biological variability of the biomarker based on the following formula:

$$ {RR}_{\mathrm{true}}={e}^{\left({\mathrm{lnRR}}_{\mathrm{observed}}\ast \frac{1}{\mathrm{ICC}}\right)} $$

## Additional files

Additional file 1: Detection range and percentage detected for plasma cytokine levels in all samples. (DOCX 14 kb)

Additional file 2: Stratified ICCs, strata based on sample median at baseline. (DOCX 17 kb)

Additional file 3: Cytokine baseline concentrations stratified by age tertiles. (DOCX 16 kb)

Additional file 4 Overview of cytokine reliability studies in predominantly healthy individuals. (DOCX 19 kb)

Additional file 5 Observed relative risk (RR) measurements of cytokines based on single measurements. (DOCX 85 kb)

## Abbreviations

BMI: Body mass index
CI: Confidence interval
ELISA: Enzyme linked immunosorbent assay
EPIC: European Prospective Investigation into Cancer and Nutrition
hsCRP: High sensitivity C-reactive protein
ICC: Intraclass correlation coefficient
IFN-γ: Interferon gamma
IL: Interleukin
MSD: Meso Scale Discovery
TNF-α: Tumor necrosis factor alpha
WC: Waist circumference
