# Supplementary material for: Cytokines for evaluation of chronic inflammatory status in ageing research: reliability and phenotypic characterisation
Source: Immun Ageing. 2019 May 21;16:11. doi: 10.1186/s12979-019-0151-1 (PMC6530020; doi:10.1186/s12979-019-0151-1)
Supplement: Supplementary file 1 — Detection range and percentage detected for plasma cytokine levels in all samples. (DOCX 14 kb) [file 12979_2019_151_MOESM1_ESM.docx]

**Additional file 1.** Detection range and percentage detected for plasma cytokine levels in samples collected twice over 4 months

| Cytokines (pg/ml) | LLOD range^a^ | % detected^b^ |
| --- | --- | --- |
| *Interleukin-1beta* | 0.01-0.27 | 38.9 |
| *Interleukin-2* | 0.01-0.29 | 66.2 |
| *Interleukin-4* | 0.01-0.05 | 71.3 |
| *Interleukin-6* | 0.01-0.11 | 100 |
| *Interleukin-8* | 0.01-0.13 | 100 |
| *Interleukin-10* | 0.01-0.15 | 100 |
| *Interleukin-12p70* | 0.02-0.89 | 95.9 |
| *Interleukin-13* | 0.03-0.73 | 80.9 |
| *Interferon-gamma* | 0.05-0.62 | 100 |
| *Tumor Necrosis Factor alpha* | 0.01-0.13 | 100 |

^a^Lower limit of detection is a calculated concentration corresponding to the signal 2.5 standard deviations above the zero calibrator, based on 38 runs from MSD Cytokine Assay protocol (Rockville, Maryland, USA), ^b^Average % detected at both time points in our study participants.
Abbreviations: ICC, intraclass correlation coefficient; LLOD, lower limit of detection
