# Supplementary material for: Cytokines for evaluation of chronic inflammatory status in ageing research: reliability and phenotypic characterisation
Source: Immun Ageing. 2019 May 21;16:11. doi: 10.1186/s12979-019-0151-1 (PMC6530020; doi:10.1186/s12979-019-0151-1)
Supplement: Supplementary file 2 — Stratified ICCs, strata based on sample median at baseline. (DOCX 17 kb) [file 12979_2019_151_MOESM2_ESM.docx]

**Additional file 2.** Stratified ICCs, strata based on sample median at baseline.

|  | **BMI**  **( kg/m^2^)** | | **Waist circumference (cm)** | | **hsCRP**  **(ug/ml)** | | **Age**  **(years)** | |
| --- | --- | --- | --- | --- | --- | --- | --- | --- |
|  | Below median | Above median | Below median | Above median | Below median | Above median | Below median | Above median |
| *IL-1b* |  |  |  |  |  |  |  |  |
| ICC | 0.08 | 0.35 | 0.18 | 0.29 | 0.11 | 0.45 | 0.21 | 0.26 |
| 95% CI | -0.29, 0.40 | 0.05, 0.58 | -0.19, 0.48 | -0.01, 0.53 | -0.20, 0.38 | 0.11, 0.69 | -0.14, 0.49 | -0.06, 0.52 |
| *IL-2* |  |  |  |  |  |  |  |  |
| ICC | 0.32 | 0.25 | 0.27 | 0.29 | 0.31 | 0.21 | 0.36 | 0.21 |
| 95% CI | 0.08, 0.52 | 0.01, 0.45 | 0.03, 0.48 | 0.06, 0.49 | 0.10, 0.48 | -0.08, 0.46 | 0.13, 0.55 | -0.03, 0.42 |
| *IL-4* |  |  |  |  |  |  |  |  |
| ICC | -0.06 | -0.30 | -0.03 | -0.34 | -0.08 | -0.30 | -0.19 | -0.19 |
| 95% CI | -0.30, 0.17 | -0.50, -0.09 | -0.26, 0.20 | -0.54, -0.13 | -0.29, 0.12 | -0.53, -0.05 | -0.40, 0.03 | -0.41, 0.04 |
| *IL-6* |  |  |  |  |  |  |  |  |
| ICC | 0.60 | 0.51 | 0.58 | 0.55 | 0.55 | 0.65 | 0.64 | 0.55 |
| 95% CI | 0.46, 0.71 | 0.35, 0.64 | 0.44, 0.70 | 0.40, 0.67 | 0.42, 0.66 | 0.50, 0.76 | 0.51, 0.74 | 0.40, 0.67 |
| *IL-8* |  |  |  |  |  |  |  |  |
| ICC | 0.58 | 0.66 | 0.58 | 0.67 | 0.62 | 0.65 | 0.57 | 0.68 |
| 95% CI | 0.43, 0.69 | 0.54, 0.76 | 0.44, 0.69 | 0.55, 0.76 | 0.50, 0.71 | 0.50, 0.76 | 0.43, 0.69 | 0.56, 0.77 |
| *IL-10* |  |  |  |  |  |  |  |  |
| ICC | 0.71 | 0.50 | 0.66 | 0.57 | 0.68 | 0.50 | 0.65 | 0.57 |
| 95% CI | 0.60, 0.79 | 0.34, 0.63 | 0.54, 0.76 | 0.43, 0.69 | 0.57, 0.76 | 0.32, 0.65 | 0.52, 0.75 | 0.43, 0.69 |
| *IL-12p70* |  |  |  |  |  |  |  |  |
| ICC | 0.39 | 0.24 | 0.47 | 0.16 | 0.35 | 0.27 | 0.47 | 0.17 |
| 95% CI | 0.21, 0.54 | 0.05, 0.42 | 0.30, 0.61 | -0.04, 0.35 | 0.18, 0.49 | 0.05, 0.47 | 0.30, 0.61 | -0.03, 0.35 |
| *IL-13* |  |  |  |  |  |  |  |  |
| ICC | 0.36 | 0.43 | 0.39 | 0.40 | 0.34 | 0.56 | 0.35 | 0.44 |
| 95% CI | 0.15, 0.53 | 0.24, 0.59 | 0.19, 0.55 | 0.20, 0.57 | 0.16, 0.50 | 0.35, 0.71 | 0.15, 0.52 | 0.24, 0.60 |
| *IFN-γ* |  |  |  |  |  |  |  |  |
| ICC | 0.29 | 0.50 | 0.25 | 0.50 | 0.36 | 0.44 | 0.32 | 0.45 |
| 95% CI | 0.10, 0.45 | 0.34, 0.63 | 0.07, 0.43 | 0.34, 0.63 | 0.20, 0.50 | 0.24, 0.61 | 0.14, 0.49 | 0.28, 0.59 |
| *TNF-* *α* |  |  |  |  |  |  |  |  |
| ICC | 0.76 | 0.61 | 0.77 | 0.60 | 0.62 | 0.80 | 0.64 | 0.76 |
| 95% CI | 0.66, 0.83 | 0.48, 0.72 | 0.68, 0.84 | 0.46, 0.71 | 0.50, 0.72 | 0.70, 0.87 | 0.51, 0.74 | 0.67, 0.83 |

Medians: BMI, 26.1 kg/m^2^; waist circumference, men 100.8 cm, women 86.3 cm; hsCRP, 1.2 ug/mL; age, 56.7 years.
Abbreviations: BMI, body mass index; CI, confidence interval; CRP, c-reactive protein; ICC, intraclass correlation coefficient; IL-1β, interleukin-1beta; IL-2, interleukin 2; IL-4, interleukin 4; IL-8, interleukin 8; IL-10, interleukin 10; IL-12p70, interleukin 12p70; IL-13, interleukin 13; IFN-γ, interferon gamma; IQR, interquartile range; TNF- α, tumor necrosis factor alpha

Overall, these analyses did not reveal apparent differences according to strata.
