# Supplementary material for: Cytokines for evaluation of chronic inflammatory status in ageing research: reliability and phenotypic characterisation
Source: Immun Ageing. 2019 May 21;16:11. doi: 10.1186/s12979-019-0151-1 (PMC6530020; doi:10.1186/s12979-019-0151-1)
Supplement: Supplementary file 3 — Cytokine baseline concentrations stratified by age tertiles. (DOCX 16 kb) [file 12979_2019_151_MOESM3_ESM.docx]

**Additional file 3.** Cytokine baseline concentrations stratified by age tertiles

| *Cytokines (pg/ml)* | Age tertile categories (years) |  | |  |
| --- | --- | --- | --- | --- |
|  |  | N | Median (IQR) | *P* difference^a^ |
| *Interleukin-1beta* | 44.9–54.7 | 25 | 2.7 (0.7-7.4) |  |
|  | 54.7–58.8 | 26 | 2.4 (0.1-5.8) | 0.31 |
|  | 58.8 – 64.0 | 28 | 3.5 (1.5-6.7) |  |
|  |  |  |  |  |
| *Interleukin-2* | 44.9–54.7 | 46 | 0.5 (0.2-4.5) |  |
|  | 54.7–58.8 | 45 | 3.8 (0.4-6.8) | 0.05 |
|  | 58.8 – 64.0 | 52 | 1.7 (0.2-6.4) |  |
|  |  |  |  |  |
| *Interleukin-4* | 44.9–54.7 | 47 | 2.5 (1.5-5.5) |  |
|  | 54.7–58.8 | 50 | 2.9 (1.5-6.3) | 0.82 |
|  | 58.8 – 64.0 | 46 | 2.8 (1.7-5.6) |  |
|  |  |  |  |  |
| *Interleukin-6* | 44.9–54.7 | 68 | 0.5 (0.4-0.7) |  |
|  | 54.7–58.8 | 70 | 0.5 (0.4-0.8) | 0.04 |
|  | 58.8 – 64.0 | 69 | 0.6 (0.4-0.8) |  |
|  |  |  |  |  |
| *Interleukin-8* | 44.9–54.7 | 68 | 3.5 (2.8-4.8) |  |
|  | 54.7–58.8 | 70 | 3.8 (3.2-4.9) | 0.07 |
|  | 58.8 – 64.0 | 69 | 4.2 (3.3-4.9) |  |
|  |  |  |  |  |
| *Interleukin-10* | 44.9–54.7 | 68 | 0.2 (0.2-0.3) |  |
|  | 54.7–58.8 | 70 | 0.3 (0.2-0.4) | 0.23 |
|  | 58.8 – 64.0 | 69 | 0.3 (0.2-0.3) |  |
|  |  |  |  |  |
| *Interleukin-12p70* | 44.9–54.7 | 65 | 0.2 (0.1-5.3) |  |
|  | 54.7–58.8 | 69 | 1.5 (0.2-7.2) | 0.11 |
|  | 58.8 – 64.0 | 60 | 0.3 (0.1-4.3) |  |
|  |  |  |  |  |
| *Interleukin-13* | 44.9–54.7 | 54 | 0.7 (0.5-1.0) |  |
|  | 54.7–58.8 | 60 | 0.7 (0.4-0.9) | 0.82 |
|  | 58.8 – 64.0 | 53 | 0.6 (0.4-1.0) |  |
|  |  |  |  |  |
| *Interferon-gamma* | 44.9–54.7 | 68 | 2.9 (2.2-4.2) |  |
|  | 54.7–58.8 | 70 | 3.0 (2.2-4.3) | 0.68 |
|  | 58.8 – 64.0 | 69 | 3.3 (2.0-5.0) |  |
|  |  |  |  |  |
| *Tumor Necrosis Factor alpha* | 44.9–54.7 | 68 | 1.8 (1.4-2.2) |  |
|  | 54.7–58.8 | 70 | 2.0 (1.7-2.4) | 0.06 |
|  | 58.8 – 64.0 | 69 | 2.0 (1.6-2.4) |  |
|  |  |  |  |  |

^a^*P* values based on Wilcoxon rank sum test (Kruskal-Wallis)
