# Supplementary material for: Cytokines for evaluation of chronic inflammatory status in ageing research: reliability and phenotypic characterisation
Source: Immun Ageing. 2019 May 21;16:11. doi: 10.1186/s12979-019-0151-1 (PMC6530020; doi:10.1186/s12979-019-0151-1)
Supplement: Supplementary file 4 — Overview of cytokine reliability studies in predominantly healthy individuals. (DOCX 19 kb) [file 12979_2019_151_MOESM4_ESM.docx]

**Additional file 4.** Overview of cytokine reliability studies in predominantly healthy individuals

| Reference | Assay | Sample | N | Gender | Age in years^a^ | Measurement points | Time between data collection |
| --- | --- | --- | --- | --- | --- | --- | --- |
| McKay et al. 2017 | MSD | Serum | 250 | Men | N/A | 2 | After 0-2 years |
| Belzeaux et al. 2017 | MSD and Luminex | Plasma | 10 | Men, Women | N/A | 4 | After 2, 8, and 30 weeks |
| Epstein et al. 2013 | Luminex | Serum | 200 | Men | 34.5 (21.6-54.6) | 3 | Every 12 months |
| Todd et al. 2013 | Erenna | Plasma | 25 | Men, Women | 36 (26-61) | 6 | Every week |
| Nash et al. 2013 | ELISA | Serum | 36 | Men, Women | N/A | 2 | After 10 months |
| Navarro et al. 2012 | ELISA/  Luminex | Serum | 62 | Men, Women | 30.4 ± 6.1 | 4 | Every 6 weeks |
| Hofmann et al. 2011 | Luminex | Serum | 28 | Men, Women | Mean: 61 (55-70) | 3 | After 1 and 5 years |
| Clendenen et al. 2010 | Luminex | Plasma | 18 | Women | 78.9 | 2 | After 1-3 years |
| Gu et al. 2009 | Luminex | Serum | 65 | Women | 50.8 ± 8.2 | 3 | Every 12 months |
| ^b^Lee et al. 2007 | Luminex | Serum | 48 | Men | 54.8 ± 9.19 | 4 | Every 3 months |

^a^Age is represented as mean ± SD or median (range). ^b^ In this study 8% of study participants had diabetes or atherosclerosis
Abbreviations: ELISA: enzyme-linked immunosorbent assay; MSD: Meso Scale Discovery electrochemiluminescence platform, Luminex: bead-based multiplex assay
Studies excluded who measured cytokines at one time point in duplicate (22, 23), or only including young women (age 19.5 ± 3.2 years) (25)
