# Supplementary material for: Cytokines for evaluation of chronic inflammatory status in ageing research: reliability and phenotypic characterisation
Source: Immun Ageing. 2019 May 21;16:11. doi: 10.1186/s12979-019-0151-1 (PMC6530020; doi:10.1186/s12979-019-0151-1)
Supplement: Supplementary file 5 — Observed relative risk (RR) measurements of cytokines based on single measurements. (DOCX 85 kb) [file 12979_2019_151_MOESM5_ESM.docx]

**Additional file 5.** Observed relative risk (RR) measurements of cytokines based on single measurements


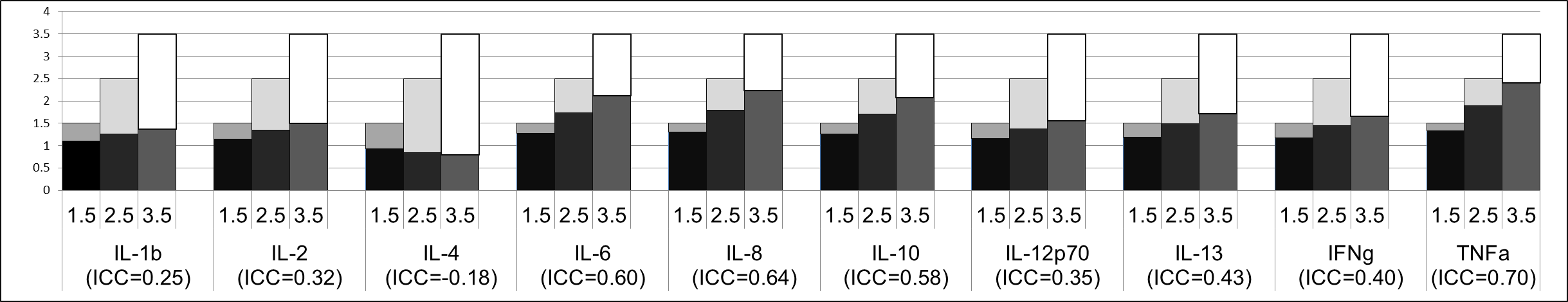


RR

Abbreviations: ICC, intraclass correlation coefficient; IL-1β, interleukin-1beta; IL-2, interleukin 2; IL-4, interleukin 4; IL-8, interleukin 8; IL-10, interleukin 10; IL-12p70, interleukin 12p70; IL-13, interleukin 13; IFN-γ, interferon gamma; IQR, interquartile range; TNF- α, tumor necrosis factor alpha; RR, relative risk.

Bars represent the observed RR based on single measurements calculated from a hypothetical true RR (1.5, 2.5, and 3.5) adjusted by the corresponding ICC for each cytokine. Discrepancies between observed and true RR are due to biological variability of the cytokine and the resulting attenuation of the exposure-outcome association. Darker shades in each bar signify observed association, brighter shades signify proportion of attenuated association through reduced reliability (based on formula: ${RR}_{true}=e^{({lnRR}_{observed}*\frac{1}{ICC})}$).
